# Heavy Metals in the Fish *Tenualosa ilisha* Hamilton, 1822 in the Padma–Meghna River Confluence: Potential Risks to Public Health

**DOI:** 10.3390/toxics9120341

**Published:** 2021-12-08

**Authors:** Md. Jahangir Sarker, Md. Ariful Islam, Farhana Rahman, Md. Anisuzzaman

**Affiliations:** Department of Fisheries and Marine Science, Noakhali Science and Technology University, Noakhali 3814, Bangladesh; fisharif34@gmail.com (M.A.I.); ananya.fr92@gmail.com (F.R.); anisnstu@gmail.com (M.A.)

**Keywords:** *Tenualosa ilisha*, bioaccumulation, carcinogenic risk, human health, non-carcinogen

## Abstract

Hilsa shad (*Tenulosa ilisha*) is Bangladesh’s most important single-species fishery that contributes to 11% of total catch and employment for millions of people. However, heavy metals (HMs) toxicity in the edible organs of *T*. *ilisha* and their plausible public health threats have received weak attention. To provide insights on this issue, we determined, using ICP-MS, the concentration of Zn, Cu, Cr (VI), Pb, and Cd in the edible organs of five different sizes of *T*. *ilisha* and the surface water collected from the Padma–Meghna River confluence, Chandpur (Bangladesh). Multivariate analysis indicated that *T*. *ilisha* gills and liver contained higher HMs than muscle, and the surface water was below the safety limits. The study revealed that only Cr crossed the safety limits and bioaccumulated in the smaller-sized gills and liver. To assess the public health risks, target hazard quotient (THQ), total THQ (TTHQ) and carcinogenic (CR) risks were calculated. Only Cr imposed non-carcinogenic risks to consumers, while TTHQ showed higher chronic health risks. There was no CR risk measured for consumers, except for the largest-sized gills for children. Randomly positive relations between HMs and sizes were found; whereas, consistently positive relations were found among the tissue types. The outcomes of our study may aid policymakers in managing pollutants, especially the Cr sources in the greater Chandpur regions.

## 1. Introduction

The hilsa shad (*Tenulosa ilisha*, Hamilton, 1822) is one of the largest commercial species of fish in Bangladesh. This single-capture fishery is common in almost all major River ecosystems, including the Padma, Meghna, and Jamuna Rivers, their estuaries, and the Bay of Bengal [1]. The highest *T*. *ilisha* catch is typically landed in Bangladesh waters (60% of the total catches), followed by Myanmar (20%) and India (15%), while the remaining 5% is landed in other neighboring regions [2,3]. In Bangladesh, the fishery of the *T*. *ilisha* contributes 1% of the GDP and 12% of the total national fish production, which represents a 65% share of the marine fish capture in Bangladesh [1]. In line with the recent increases in production, heavy metals (HMs) and metal-based pollutants in the aquatic environment have drawn major attention in the fishery industry [4]. A broad range of toxic HMs origins, such as anthropogenic activities like industrial, urban and residential, agricultural, catchment runoff, shipping, and mining [5,6], ultimately follow waterways to assimilate and bioaccumulate in fishes, generating health risks to humans [7]. The HMs pathway is reaching humans through the food chain, i.e., industry–topsoil–catchment–plankton–fish–human. HMs concentration in water mirrored the concentrations in fish gills, resulting in an indicator of habitat status [8]. Gills usually carry higher levels of HMs than muscles, and HMs are stored in the liver as metallothioneins group [8]. Because gills always come into open contact with water, exchange of respiratory gases, balance osmoregulation, nitrogen excretion, and importantly gill surface are negatively charged, which can bind the positively charged HMs ion [9,10,11]. However, the HMs concentration in fish varies with age, different trophic levels, habitats, etc. [8].

Although HMs such as Zn, Cu, and Cr perform several biochemical and physiological functions and oxidation-reduction reactions in animals, excess amounts of HMs are toxic to humans and other animals even at much lower concentrations [12,13,14]. For example, excess intake of the recommended daily allowance (RDA as mg day^−1^) of Zn (8.0 female, 11.0 male), Cu (1.0 children, 10.0 adults), Cr (VI) (0.035) (Pb 0.005), and Cd (0.025), respectively, may cause several health risks [13,14,15]. For example, chronic exposure to Pb may cause renal failure and liver dysfunction [14,16,17]; and severe exposure may cause coma, mental obstacles, or death [18]. Likewise, naturally occurring Cr is found in the form of Cr (II) to Cr (VI). Predominantly, Cr (VI) is released from industrial establishments and anthropogenic sources and occurs naturally in groundwater and surface water [14,19]. Cr helps in glucose metabolism, but deficiency may obstruct growth and influence protein, lipid, and carbohydrates metabolism [20]. Nonetheless, in severe cases, Cr(VI) can cause respiratory, cardiovascular, gastrointestinal, hematological, renal, and neurological dysfunctions [21] and damage to the liver, lungs, and kidneys [19,22]. Both Cu and Zn are indispensable components and beneficial to health as they support hemoglobin formation, carbohydrate metabolism, and cytochrome-c oxidase [19]. However, excessive intake of HMs may cause coronary heart disease to increase plasma cholesterol [20].

In Bangladesh, the Padma–Meghna freshwater River system is one of the important breeding hotspots for anadromous *T*. *ilisha* [23]. The brood *T*. *ilisha* migrates from marine water to these Rivers for breeding. Later, they spend their nursery and juvenile stages in freshwater and estuarine conditions. The most popular sizes of the *T. ilisha* consumed from the Padma–Meghna freshwater River system in Bangladesh varied from 500–1000 g [24]. Consumers are primarily interested in the edible portion of the fish, which is the flesh or muscle, but gills are often consumed with the entire head and liver individually. Besides, the fish meal producers are concerned with the whole fish, but the fish processor is looking for the liver to prepare oil. However, these riparian ecosystems are being polluted by several sources (above mentioned) and consumer safety has become a major issue. Although several studies have been reported on HMs and possible health risks in Bangladesh in *T*. *ilisha* and other fishes [6,25,26,27,28,29,30,31,32], none of the studies correlated the HMs concentrations concerning body size. HMs content in freshwater fishes (*Channa striatus*, *Clupisoma garua*, *Glossogobius giuris* and *Heteropneustes fossilis*) organs such as liver, gills, and muscles were determined in the Meghna River, Gazaria Upazila (near Dhaka city) [33] and the Buriganga River, Dhaka, Bangladesh, respectively [34]. HMs concentrations in *T*. *ilisha* and other commercially important fishes have also been documented from both Bangladesh [35] and Indian Sundarbans mangrove [36], respectively. In addition, HMs contents in water and sediments were documented in the Meghna River [37,38], Buriganga River [39], and surface water of Bay of Bengal (Bangladesh) [40]. Some authors have studied HMs concentration in *T*. *ilisha*, and other commercially important fishes from the Gangetic delta and coastal West Bengal (India) [41], Ganga basin [42], Indian Bay of Bengal [43], Myanmar [44], Iraqi waters [45], Shatt Al-Arab River [46], and Malaysia [7]. Al-Najare et al. [45,46] reported HMs concentration in several organs of *T*. *ilisha*, such as liver, gonads, gills, intestine, and muscles. Furthermore, HMs concentration in fishes concerning their body size was documented in China [47] and north of Persian Gulf (Iran) [48]. However, none of those studies addressed the possible relationships between fish size and HMs concentration in different edible organs, like muscles, liver, and gills of *T*. *ilisha*. To provide insights on this gap of knowledge, we: (1) determined the concentrations of five HMs [Zn, Cu, Pd, Cd, and Cr (VI)] in different size groups (S1–S5) of *T*. *ilisha* and surface water; (2) estimated the relationships between the investigated HMs and *T*. *ilisha* biometric parameters (i.e., length and weight); (3) measured HMs concentration in the muscle, liver, and gills of *T*. *ilisha*; and (4) calculated the bioaccumulation factors, and the possible noncarcinogenic and carcinogenic human health risks, using correlative and multivariate statistical approaches.

## 2. Materials and Methods

### 2.1. Study Area and Sample Collection

**Figure 1:**
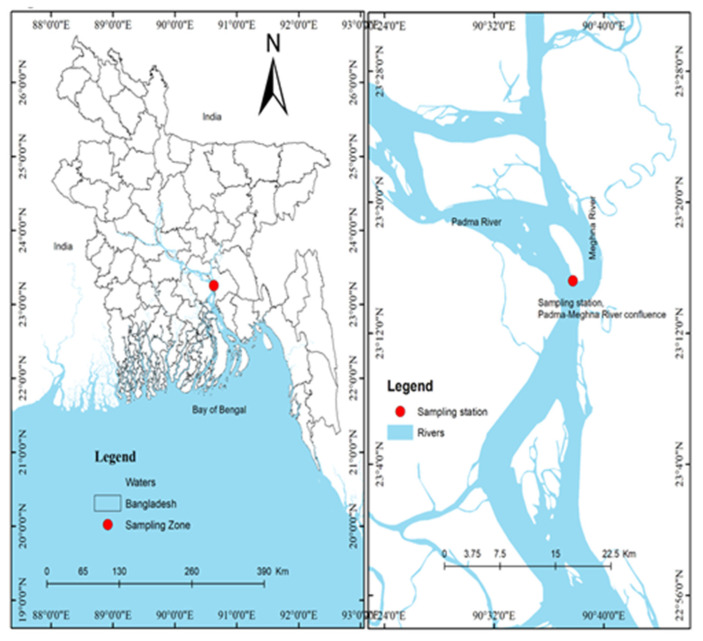
Study area and station location at the Padma–Meghna River confluence.

The Meghna River originates in the Kishoregonj District of Barak River, India, and enters the sea in Bangladesh. The Padma (Ganges) River also originates in India, then discharges at Shibgonj in the Chapai Nababganj district in Bangladesh. Combined, the flow of the Padma and the Brahmaputra (Jamuna) River was the same as that of the Padma River. Subsequently, the water flow confluences at the Meghna River in Chandpur district and is then diluted into the Bay of Bengal, Bangladesh (Figure 1).

Both the Padma and Jamuna River confluence receive around 85% water flow from the North-West latitude, while the residual 15% flow is received from the Meghna River from the North-East latitude of Bangladesh [49]. Annually, the upstream receives 3000–4900 mm and the downstream receives 1500–2400 mm rainfall. Usually, the upstream precipitates 52% (1600–2500 mm) and the downstream precipitates 60% (900 mm to 1500 mm) rainfall during June to August [49]. Globally, the highest amount of sediment and the third-highest amount of water is discharged by the Meghna River confluence [49]. This is the widest (12 km downstream) river with 264 km long, having 82,000 km^2^ of total catchment area [37]. The average and maximum depth of the Meghna river is 308 m and 490 m, while the Padma river’s average and maximum depth are 295 m and 479 m, respectively. This river ecosystem has been recognized as one of the weighty nurseries and breeding grounds for *T*. *ilisha* in Bangladesh [26]. Various sizes of *T*. *ilisha* were caught using gill nets by local fishers, and 12 water samples (four of each at < 1.0, ~ 5.0, and < 10.0 m depth, respectively) were collected using a Van Dorn water sampler in August (2019) at the Padma–Meghna River watershed and the surrounding areas of the Chandpur district (Figure 1). A total of 300 *T*. *ilisha* specimens were iced and packed in an air-tight insulated box. The water samples were poured into previously labeled, high density polyethylene bottles (4.0 L capacity), HNO_3_ (10.0%) was added, and samples were rinsed repeatedly with deionized water. Then, all samples were taken to the laboratory of the Department of Fisheries and Marine Science, Noakhali Science and Technology University for analysis.

### 2.2. HMs Analysis in Fish

*T. ilisha* specimens were stored at −22 °C and thawed before analysis. The specimens were sorted and divided into five different size groups (consisting of 25 specimens in each group) as described in Table 1. Measuring tape and digital balance (YY-768, Xpart, RFL, Bangladesh) were used to measure the total body length (L, cm), standard length (SL, from the tip of the snout (mouth closed) to the beginning of the caudal fin, cm), and weight (g) of *T. ilisha*. A sterile sharp knife and forceps were used to dissect fish, and the guts were extracted from the intestines. The liver and gills were separated from each of the fish group (*n* = 25) and labeled properly. Then, 2.0 g of wet tissues (muscles (*n* = 25), gills (*n* = 25) and ≤ 2.0 g of liver (*n* = 25, whole liver was considered those were < 2.0 g)) from each size group were freeze-dried in a VacCo 2 series freeze drier (Zirbus, Germany; condenser volume, 5.7 L; capacity 2 kg d^−1^). The freeze-dried samples (*n* = 75 from each group) were transferred to acidic water, washed in a porcelain mortar, ground into a fine powder using a pestle, and frozen at −14 °C until analysis. Using a digital electrical balance, 0.25 mg of freeze-dried samples (*n* = 75) from each size group were weighed (Model: PS.P3.310, P-Scale, Taiwan) accurately. Digestive reagents, 5 mL of deionized water, 5 mL of ultra-pure nitric acid (65% HNO_3_), and 2 mL of hydrogen peroxide (H_2_O_2_, 30%), were prepared. The digestive reagents and weighed tissues were then placed in the digestion vessels. Then, the vessels containing samples were mixed for 5 min in a vortex mixer (2000 rpm, Mod. HS120214, Heathrow Scientific), and subjected to microwave digestion (1000 W, Berghof-MWS2, Berghof speed wave, Eningen, Germany) by the following program: 10 min, 180 °C, 800 W; followed by 10 min, 190 °C, 900 W; and finally, 10 min, 100 °C, 400 W. After digestion, the mixer was filtered through Whatman paper (0.42 μm pore size) and transferred into a Teflon tube. Milli-Q water was filled into the tube up to a volume of 50 mL and then transferred and stocked into 50 mL polypropylene centrifuge tubes (Nalgene, New York, NY, USA).

All the samples were analyzed by ICP-MS (ELAN9000, Perkin-Elmer, Rodgau, Germany). The calibration was standardized by a multi-component standard (ELAN 9000/6X00, TruQ^™^ms, Perkin-Elmer, Rodgau, Germany) solution. Before beginning the analysis, the relative standard deviation (RSD of <5%) was verified by the calibration solution (20.0 µg mL^−1^: Cd, Cu, Pb, Mg, Rh, 1% HNO_3_) purchased from Perkin-Elmer. Internal calibration standard solutions containing 0.5 µg g^−1^ of each indium (In), yttrium (Y), cobalt (Co), and thallium (TI) were also purchased from Perkin-Elmer. The working standards (0, 10, 20, 50, and 100 µg g^−1^) were made by diluting the multi-component stock solution. Detection limits for Cr (VI) and Pb were set at 0.0001 µg g^−1^, while, Cd, Zn and Cu were 0.00002, 0.01, and 0.001 µg g^−1^, respectively. A test batch was counted only if the value fulfilled the given internal calibration point. For every batch analysis, one blank sample and one verified reference material [NMIJ CRM 7402-Cod fish tissue for Zn (23.3 ± 3.50, mean ± SD, µg g^−1^, dry weight)] were measured by ICP-MS. In addition, selected specimens were measured in duplicate to avoid batch-specific errors. The highest average recovery (%) was measured in Cr (VI) (110) followed by Pb (105), Cd (104), Zn (98), and Cu (96), respectively. The estimated concentrations of heavy metals in *T*. *ilisha* were expressed in µg g^−1^. In the cited literature and guidelines HMs concentration measured in wet weight (wt.) was converted into dry wt. with assuming an average of 74% water present in tissues [50] and presented within bracket as dry. wt. (wet wt.) µg g^−1^.

### 2.3. HMs Analysis in Water

ICP-MS (model: same as above) was used to determine the Cr, Cu, Zn, Pb, and Cd concentrations in water samples. Each 100 mL water sample (a total of 12 samples from < 1 to <10.0 m depth) was collected in a beaker and digested in 2.0% HNO_3_ (ultra-pure). The digested samples were filtered through Whatman filter paper (0.42 μm pore size) and then transfer in a Teflon tube. Milli-Q water was filled into the tube up to a volume of 50 mL and then transferred and stored in 50 mL polypropylene centrifuge tubes (Nalgene, New York). The relative standard deviation (RSD of <5%) was verified and 10.0 µg mL^−1^ multi-standard calibration solution (purchased from Perkin-Elmer) was prepared for all samples. In addition, a standard 1.0 mg mL^−1^ Pb (Lead standard 5% HNO_3_, matrix, Perkin-Elmer) was performed for accurate multi-element calibration. The working standards (0, 10, 20, 50, and 100 µg mL^−1^) were made by diluting the multi-component stock solution. Detection limits for Cr (VI), Pb, Cd, Zn and Cu were set 0.00004, 0.000017, 0.00005, 0.00016, and 0.000036 µg mL^−1^, respectively. For every batch experiment, one blank sample and a drinking water reference material (NIST^®^ CRM 1643e, Perkin-Elmer) for Zn (spiked 0.08 ± 0.002 µg mL^−1^) were used for the precision of the method. The highest average recovery (%) was measured in Cr (VI) (108.6) followed by: Pb (103.8), Cd (101.6), Zn (99.4) and Cu (99.1), respectively. The estimated HMs concentrations in water were expressed in µg mL^−1^.

### 2.4. Estimate of Potential Risks to Human Health

The Target Hazard Quotient (THQ) was analyzed as a fresh weight to measure the possible health threats due to humans’ exposure to HMs and calculated with the following equation [6,51]:

THQ = (Mc × IR × 10^−3^ × EF × ED)/(RfD × BW × ATn) (1)

where: Mc denotes the metal concentrations in *T*. *ilisha* (µg g wet weight^−1^); IR indicates the daily consumption rate in Bangladesh (g day^−1^; 52.5 g day^−1^ and 55.5 g day^−1^, fresh weight for children and adults, respectively [26]; EF (based on a 7 d week^−1^ basis) is the annual exposure frequency (days year^−1^); ED is the life exposure duration (over a 65-years mean human lifetime) [52]; RfD is the oral reference dose (mg kg^−1^ person^−1^ d^−1^): 0.003, 0.04, 0.3, and 0.001 for Cr(VI), Cu, Zn and Cd, respectively [53]; U.S. Environmental protection agency (EPA) did not provide the RfD value for Pb in food items. It is under discussion, we considered the value from Hang et al. [54], i.e., 0.0035 mg kg^−1^ person^−1^ d^−1^.

BW is the average body weight for children (15 kg) and adults (65 kg) [52]; ATn is the age in years, e.g., 65 years (23,725 days). If THQ < 1, there is no noncarcinogenic risk [55]; if THQ ≥ 1, there is a potential health risk, and protections and safety measures are to be taken.

TTHQ is the total non-carcinogenic health risks (TTHQ) imposed by several HMs. It is calculated by the summation of THQ values of the selected HMs in *T*. *ilisha* tissues for both adults and children [56]:

TTHQ = total THQs = THQ (for Cr) + THQ (for Cu) + THQ (for Zn) + THQ (for Pb) + THQ (for Cd)(2)

The TTHQ > 1 indicates chronic health threat from the total tissues of *T*. *ilisha*. 0.1 ≤ TTHQ <1 = low; 1 ≤ TTHQ < 4 = medium, and TTHQ ≥ 4 = high [7,57].

Carcinogenic risk (CR) was calculated to identify the possibility of cancer occurring in humans over a lifespan of exposure to carcinogens [58]. The accepted range of CR is between 10^−6^–10^−4^ (the risk of developing cancer is 1 in 1,000,000 to 1 in 10,000 over the average human lifespan) [59,60]. If CR > 10^−4^, there have a possible CR risk [61,62]). The CR is calculated by multiplying the carcinogenic slope factor of the HMs [63] as shown in Equation (3) [64]:

CR = (Mc × IR × 10^−3^ × CPSo × EF × ED)/(BW × ATn) (3)

where CPSo is the oral slope factor of carcinogens (mg kg^−1^ day^−1^)^−1^ taken from the Integrated Risk Information System supplied by the USEPA [61]. As Cu and Zn are not carcinogenic and Cr is mutagenic, CPSo values were found for Cd (6.3) [65,66] and Pb (0.0085) value only [51,67]. The possibility of forming cancer for a consumer will be > 1 in 100,000 when CR values exceed 10^−5^ [6,51].

The bioaccumulation factor (BAF) indicates the abundance of trace metals (µg g wet weight^−1^) that have accumulated in the organs of *T*. *ilisha* [58]. BAF is calculated by determining the difference in the HMs accumulation in *T*. *ilisha* tissues and in the aquatic environment [68], as follows:

BAF = HM_hilsa_/HM_water_ (4)

where HM_hilsa_ represents the HMs concentration in *T*. *ilisha* (µg g^−1^ wet wt.) and HM_water_ represents the HMs concentrations in the water specimens (µg mL^−^^1^). Due to the HMs equivalent units in fish tissues and water, BAFs have no units. BAFs are classified into the following ranges: BAF <1000 = low possibility of accumulation; 1000 ≤ BAF ≤ 5000 = bio-accumulative; and BAF > 5000 = highly bio-accumulative [26].

### 2.5. Length-Weight (L-W) Relationships and Condition Factor in T. ilisha

In total, a subsample of 125 *T*. *ilisha* specimens were measured and weighed and divided into five size classes (S1–S5; Table 1). L-W relationships of *T*. *ilisha* were determined according to the following equation [69]:

W = a × L^b^ (5)

where, W is the total weight of *T*. *ilisha* (g), L is the total length (cm); a is the intercept of the regression, and b is the regression coefficient (slope). If the b = 3, it indicates isometric growth, b > 3 indicates positive allometric growth, and b < 3 indicates negative allometric growth.

The condition factor (CF) was measured according to Froese [70] as follows:

CF = 100 × W/(SL)^3^ (6)

where SL is the standard length of *T*. *ilisha* from the Padma–Meghna Rivers confluence. CF > 1.0 indicates healthy growth, and CF < 1.0 indicates non-healthy growth of *T*. *ilisha*.

### 2.6. Data Analysis

Univariate and multivariate statistical analyses were performed in the SPSS software (IBM, Version: 23.0) and PAleontological STatistics (PAST, Version: 4.02), respectively. The length-weight relationships among sizes and organs of *T*. *ilisha* were graphed and analyzed by regression, single-factor analysis of variance (one-way ANOVA). The estimated HMs and other values were presented as mean ± SD. Principal component analysis (PCA) and Pearson correlation matrix were performed in PAST.

## 3. Results

### 3.1. Fish Morphometry

The mean length of the 125 *T*. *ilisha* specimens from the Padma–Meghna Rivers’ confluence ranged from 19.1 to 46.5 cm, mean individual weight ranging from 100.1 to 1200.4 g, corresponding to <1–5 years [71,72].

An isometric (b = 3.0) L-W relationship was calculated for the whole investigated population, irrespective of size classes, whereas a negative allometric (b < 3.0) growth was determined for each of the different size classes (Table S1).

### 3.2. HMs Concentrations in T. ilisha (µg g^−1^ Dry Weight)

The average HMs concentration (± standard deviation) in the muscles, liver, and gills of each of the five size classes are reported in Table 2. HMs total concentrations in the whole *T*. *ilisha* followed the order S1 > S2 > S4 > S5 > S3. This order, however, varied in the different tissues (Table S2) and, generally, the smaller size classes (S1, S2) contained a higher HMs concentration than that in the larger size classes (S4, S5) (Table S2). Details about each of the different HMs are reported and discussed below.

#### 3.2.1. Zinc (Zn)

Zn concentration in muscle, liver, and gills in fish are presented in Table 2. The mean Zn concentration in *T*. *ilisha* was 102.71. The highest concentration of Zn (189.04) was measured in gills of size class S1, and the minimum concentration (40.88) was observed in the muscles of size class S5 (Table 2). Considering all sizes, the highest average quantities of Zn were measured 145.90 in gills, followed by the liver (108.79) and the muscles (53.45). The Zn concentration in the gills decreased gradually with an increase in the size of the *T*. *ilisha* (S1 to S3). Similar decreases (65.32 to 40.88) were observed in the muscles (S1 to S3). The Zn concentrations in the liver varied; however, the concentration was lowest in the smallest fish (S1; 63.40), and highest in the largest fish (S5; 132.76).

#### 3.2.2. Copper (Cu)

The highest concentration of Cu (25.04) was detected in gills of the larger specimens (S5), and the lowest (4.76) occurred in muscles of the S5 class. The average maximum concentrations of Cu followed the order: liver (18.50) > gills (16.08) > muscle (10.33) (Table 2). In muscles, Cu concentration was 4.76, 6.68, and 9.12 in S5, S2, and S1 size classes, respectively, whereas Cu concentrations in S3 and S4 classes exceeded 15.50 (Table 2). In the liver, Cu concentrations in the S2, S4, and, S5 size classes (23.48–24.64) were higher than those in the smallest S1 class (9.52). In gills, Cu concentration was highest in the largest S5 size class (25.04), whereas progressively increased (from 8.01 to 18. 28) with fish increasing size (from S4 to S1, respectively) (Table 2).

#### 3.2.3. Chromium (VI) [Cr (VI)]

The Cr concentration varied between 4.52 and 326.64 in the selected organs; the highest average concentration was in the gills (104.86), followed by the liver (44.87) and muscles (11.14) (Table 2). Overall, the gills of the smallest (S1) *T*. *ilisha* had the highest Cr concentration (326.64), whereas, S4 *T*. *ilisha* had the lowest concentration (23.48) (Table 2). Moreover, the livers of S2-sized *T*. *ilisha* had higher Cr concentrations (172.32) than the livers of the largest size (S5 sized) (15.90). In the case of muscles, S1-sized (7.60) and S5-sized (5.44) *T*. *ilisha* contained lower Cr concentrations than the S4-sized specimens (23.96). On average, the Cr concentration of the smallest *T*. *ilisha* (S1 size) was lower in the muscles than in the gills and liver. The average Cr content in the studied tissues followed the order as S1 > S2 > S3 > S4> S5 (Table S2).

#### 3.2.4. Lead (Pb)

The highest Pb concentration (0.086) observed was in the gills of S2-sized *T*. *ilisha*, and the lowest concentration (0.011) observed was in the muscles of S4-sized *T*. *ilisha* and S3 gills simultaneously (Table 2). The concentration of Pb in the different organs was highest in the gills (0.03), followed by the muscles (0.015) and the liver (0.014). The different sizes of *T*. *ilisha* (S1–S5) did not exhibit noticeable variations for Pb concentrations.

#### 3.2.5. Cadmium (Cd)

The concentration of Cd was the lowest (0.001) among the other HMs considered in this study. Among the examined organs, gills contained the highest concentration (0.14) of Cd, followed by the liver (0.004) and muscles (0.003). The highest Cd concentration (0.47) observed was in the gills of the largest *T*. *ilisha* (S5 size) (Table 2). In addition, the Cd concentration decreased sharply with a decrease in fish size (S4 to S3). However, no patterns we identified for the muscles or the liver (Table S2).

### 3.3. HMs Concentrations in Surface Water (µg mL^−1^)

The highest and lowest HMs concentration in the surface water were detected in Zn (0.070 ± 0.005), and Pb = Cd (0.002 ± 0.001), respectively, and followed by Cu (0.058 ± 0.04) and Cr 0.035 ± 0.002), respectively (Table 3). None of the investigated HMs in the water exceeded the recommended thresholds reported in the USEPA [33] and WHO [76,77,78] guidelines (Figure S1, Table 3). Details about each of the different HMs are reported in Table 3.

### 3.4. Noncarcinogenic (THQ), Total THQ (TTHQ) and Carcinogenic (CR) Risks

The THQ and CR determined for all size groups of *T*. *ilisha* and organs are presented in Table 4. None of the HMs crossed the accepted limit (<1), except for Cr (VI), which was enormously higher in the smallest size (S1, children-C: 99.081; adult-A: 22.445) gills of *T*. *ilisha*. The average THQ value of Cr was 16.267 and 3.685 for children and adults, respectively (Table 4). The highest mean total of THQ was calculated in gills (40.171), followed by liver (17.624) and muscles (4.641), respectively. In addition, the highest average THQ for Cr measured was as gills (31.809) > liver (13.611) > muscles (3.380) in children; likewise, it followed as gills (7.206) > liver (3.083) > muscles (0.766) in adults, respectively. Besides, the calculated TTHQ value was 254.526 and 57.658 for children and adults, respectively, indicated higher chronic health risks to the consumers. In muscles, TTHQ was 4.286 for adults; while, it was 18.921 for children, respectively. Overall, the TTHQ in the selected tissues was > 4 times higher in children (C) than adults (A). All the calculated CR values were within the limit (10^−6^–10^−4^) to either individual except for Cd in the largest size *T*. *ilisha* (S5) gills, which showed the CR risk to the children (Table 4).

### 3.5. HMs Bioaccumulation Factor (BAF)

BAF is a good indicator of HMs accumulation to the *T*. *ilisha* body from its surrounding water. The maximum bioaccumulation (2426.5) was documented in the gills of the smallest fish (S1) for Cr (VI), whereas the lowest was estimated in the gills of S1 and S3-sized fish (0.1) for Cd (Table 5). The order of the mean bioaccumulation factors (BAFs) followed as: Cr (398.4) > Zn (381.5) > Cu (67.1) > Cd (6.4) > Pb (2.5) (Table 5). Only Cr was assessed as bio-accumulative in S1 gills (2426.5) and S2 liver (1280.1), respectively.

The total sums of size-related BAFs were, S1 (3882.1) > S2 (3166.6) > S5 (1972.7) > S4 (1953.6) > S3 (1862.8). The highest total mean sums of BAFs were measured in the gills, and ranked as, gills (1414.9) > liver (822.7) > muscles (330.0), respectively.

### 3.6. Length-Weight (L-W) Relationships of T. ilisha

To assess the environmental factors, such as pollution, growth rate, feeding, and reproduction of *T. ilisha*, L-W relationships can be effective, along with its stock reviews and management [69,81,82]. The estimated standard length (SL) ranged from 16.1–36.6 cm, and the *p*-values, confidence limits (CL), coefficients of determination (*R*^2^) are provided in Table S1. A linear (*R*^2^ = 0.99) and isometric (b = 3.0) L-W correlations were observed among all the selected sizes of *T*. *ilisha*; however, a negative allometric (b < 3.0) growth was measured in the individual sizes. Overall, HMs trends ranked as S1 > S2 >S4 > S5 > S3 (Table S2). In addition, the *R*^2^ and b (slope) value followed as, S1 (0.85) > S5 (0.80) > S3 (0.69) > S4 (0.33) > S2 (0.27) and S1 (0.52) > S2 (0.32) > S3 (0.30) > S5 (0.22) > S4 (0.14), respectively (Tables S1 and S2). The hierarchy of condition factor (CF) followed as S5 (2.5) > S4 (2.4) > S1 (2.3) > S2 (2.0) > S3 (1.9) (Table S2). The HMs and b values correlated to S1 and S2 sizes (juvenile to pre-adult stages), which corresponds to ≤ 1 year [71,83].

### 3.7. Relationship between Size Groups and HMs

To investigate the HMs concentrations in relation to the body size of *T*. *ilisha*, multivariate, Pearson correlation, and linear regression were performed and presented in Figure S3 and Tables S3 and S4. Multivariate analysis, such as PCA showed that most of the tissue sizes clumped together, such as muscles (M); whereas, the liver (L) and gills (G) were dispersedly distributed in the components (Figure S2). The Pearson correlation matrix (Table S3) and linear regression (Table S4) showed that HMs had significant positive (*p* < 0.05) relations among the sizes of *T*. *ilisha* but were not consistent. S1 size muscles (S1-M) showed stronger to linear correlations (*r* = 0.96–1.0, *p* < 0.05) to their respective groups. Although S1–S2 liver was non-significant (*p* > 0.05), a linear correlations (*r* = 1.0) were found among the rest of the size groups (Table S3). Overall, a consistently significant positive (*p* < 0.05) relations were observed among the selected tissues of *T*. *ilisha* (Figure S3). In case of specific HMs vs. tissues, gills were positively significant (*p* < 0.05) to almost all HMs; while, the liver was randomly positively significant to some HMs, and muscles were always non-significant (*p* > 0.05) to the HMs. In the linear regression, none of the HMs were significant (*p* > 0.05) to its tissues in S1; while only Pb was significant (*p* < 0.05) in S2 tissues. In addition, HMs were randomly significant (*p* < 0.05) in S3, S4, and S5 tissues of *T*. *ilisha*. Among the HMs vs. size classes regression, only 21. 75% was significant (*p* < 0.05) (Table S4).

## 4. Discussion

### 4.1. HMs in T. ilisha Tissues (µg g^−1^, Dry Wt.)

In our studied materials, Zn concentration in the smallest (S1) *T*. *ilisha* was higher than the larger sizes and it followed the rank as gills > liver > and muscles. However, the measured concentration of Zn from fish muscle and organs were within the ranges as recommended by The Food and Agriculture Organization (FAO)/ World Health Organization (WHO) [73]. In addition, the average Zn content was below the guidelines provided by the California Environmental Protection Agency (CEPA) [74] and the Ministry of Fisheries and Livestock (MOFL) [75], respectively (Table 2). The Zn concentrations in the present study varied from the other riverine areas of Bangladesh [27,32,36], India [41], China [47], and Iraq [84], but were within the guidelines, except in *Amblypharyngodon mola* in the Sundarbans mangrove (Bangladesh) which exceeded all the guidelines (Table 2) [35]. Shorelines as occupied with abundant plankton due to continuous runoff, smaller sizes of *T. ilisha* might intake high content of Zn through feeding. The statement is more evident from the open area of the Bay of Bengal [31], where Zn concentration in *T. ilisha* fish exhibited more than one order of magnitude lower than the present study.

Average Cu concentration did not exceed the international guidelines (FAO/ WHO, CEPA), but the liver of the larger size (S4–S5) *T*. *ilisha* exceeded the national guideline (MOFL) (Table 2) [73,74,75]. Cu contained in the fish tissues in the present study were within the international guidelines [73]. Because of the pioneer study, heavy metal concentrations in the different size classes of *T*. *ilisha* fish are scanty in Bangladesh. Cu concentration in *T*. *ilisha* fish tissue reported in other healthy ecosystems was manifold lower than our study [26,27]. Interestingly, information on different organs of *T*. *ilisha* fish that contain metals has not yet been reported for our significant study area (Chandpur-nursery and breeding grounds) in Bangladesh. The plausible discussion, therefore, was made with other fish species. Muscles, livers, intestines, gills, and livers of native edible fishes (other than *T*. *ilisha*) in different fresh and coastal waters in Bangladesh [28,31,32,34,35,36], Iraqi marine water [45], and the Persian Gulf [48], contained Cu levels within the safety limits. However, their values varied from organ to organ and habitat changes. Gills and liver showed the highest organ-specific bioaccumulation trend compared to *T*. *ilisha* fish muscles for Cu. Cr (VI) concentration extraordinarily exceeded the national and international guidelines [73,74,75] in all sizes and tissues of *T*. *ilisha* in our study (Table 2). Gills of the smallest sizes of T. ilisha were highly prone to Cr ingestion while its rate decreased gradually with the increment of their body size except S4 size class. Cr concentrations measured from all tissues and size classes of *T*. *ilisha* in our study were several orders of magnitude higher than the studies reported from other ecological zones of the Meghna river [25,26,27]. Smaller sizes (S1, S2) of *T*. *ilisha* exposed higher Cr in their gills and livers other than muscle while S4 size contained maximum content in their muscle. Juvenile *T*. *ilisha* (herein S1; 100–180 g) might prefer a broader reliance on near-bottom areas for foraging as the study area has been recognized as one of the weighty nurseries and breeding grounds for *T*. *ilisha* in Bangladesh [23]. Due to receiving the highest sediment and the third-highest water discharges the study areas [49] might be rich in Cr content that facilitated high Cr in Juvenile *T*. *ilisha*. The most popular sizes of *T*. *ilisha* consumed ranged from 500–1000 g [24] corresponding to S4 of our selected group. The S4 size muscles contained the highest Cr content in our study. Unlike *T*. *ilisha*, other fishes in different parts of the Meghna river areas particularly polluted areas of Bangladesh also showed the extraordinary Cr content [33,35]. However, Cr content was below the detected limit from the Bay of Bengal [31], Pathorghata, Cox’s Bazar, Pirojpur, Padma, and Jamuna River (Bangladesh) [32], India [42] and China [47]. In addition, Cr crossed the guidelines in the studied fishes from Sundarbans (Bangladesh) and Indian Ganga basin [35,42] and Tigris River, Iraq [84]. Comparatively, the Bangladeshi fishes contained higher Cr (VI) than China and India.

No significant variations were observed among the different sizes (S1–S5) of fish for Pb. In all organs, the Pb concentration was far lower than the recommended limits [73,74,75] (Table 2). In Meghna estuary, Pb concentration was 3.33 µg g^-1^ in muscles of *T*. *ilisha* (24.20 cm and 184.60 g—correspond to S2 Size) [26]; however, Pb contents were measured 0.64 µg g^-1^ in the Meghna River, Narsingdi District, Bangladesh [27], and 0.62 µg g^-1^ in the Karnaphuli River [29], respectively. Pb concentration in different organs of bottom feeders *Channa striatus* (302.56–1243.23) [33] in the areas connected with a contaminated site far from our study site were 10 to 20 orders of magnitude higher than our pelagic feeder *T*. *ilisha*. However, Pb content in *T*. *ilisha* from the Bay of Bengal [31], different coastal waters of Bangladesh [32], and other native edible fishes from around our study site [6,28,34,35], India [36], Myanmar [44], Iraqi river [45], Shatt Al Arab river [46], and the Persian Gulf [48] was similar to the present findings following the limit national and international guidelines [73,74,75]. 

None of the tissues in Cd in the present study exceeded the recommended guidelines (Table 2) [73,74,75]. Besides, the Cd concentrations were within limits in *T*. *ilisha* and other freshwater fishes in the greater Meghna River, Karnaphuli River, Cox’s Bazar, Sundarbans, Bay of Bengal (Bangladesh) [6,26,27,28,29,31,35], neighboring Myanmar (corresponding to S1 group) [44], Chinese and Iraqi water [45,46,47], Persian Gulf [48], and the marine species from Sicilian coasts (Mediterranean Sea) [51]. However, Cd concentrations were more than 200 times higher in bottom feeder *Channa striatus*, *Glossogobium giuris,* and *Clupisoma garua* in the Meghna River at Gazaria Upazila, Monshigonj (near Dhaka city) than our pelagic *T*. *ilisha* [33]. In addition, Cd was higher in the greater Buriganga, Padma, Jamuna, and Paira Rivers; Coastal areas, such as Kuakata, Pathorghata, Pirojpur (Bangladesh), and Indian Sundarbans [28,32,34,36], and Tigris river [84] than our studied materials.

### 4.2. HMs in the Padma–Meghna River Water (µg mL^−1^)

HMs in the surface water at Padma–Meghna River confluence and its associated tributaries, Buriganga River (Cu, Zn, Cd), Bay of Bengal (Zn) (Bangladesh) were below the safety limits (Table 3) [37,38,39,40,76,77,78,80]. However, the studied HMs exceeded the suggested guidelines in the Ganga River (India) [42], Red Sea Coast of Jizan, Saudi Arabia [79]. In addition, Cr, Pb exceeded the guidelines in the Buriganga River [39], and Pd, Cd in the Bay of Bengal (Bangladesh), respectively [40].

### 4.3. Public Health Risk Assessment

Cr showed a noncarcinogenic threat to both consumers; while Cd imposed carcinogenic risks to the largest size gills for children (Table 4). Besides, the TTHQ value showed severe chronic health effects on children than adults. *T*. *ilisha* muscles showed the least noncarcinogenic risks than liver and gills. Comparatively, the children had > 4 times higher noncarcinogenic health risks than the adults. Chronic exposure to Cd may alter the pulmonary function and gastrointestinal irritant, decrease mineral density in bone, and cause osteoporosis [13,14,19,22]. In acute exposure, it may cause stomach illness, nausea, vomiting, muscle cramps, etc. [22]. The THQ and CR values in *T*. *ilisha* and other fishes did not show any health risks to humans in the upper Meghna River [6], Meghna estuary [26], different wholesale markets in Dhaka city [30], Ganga River (India) [42], and Sicilian coasts (Mediterranean Sea) [51]. In the Karnaphuli River (Bangladesh) *Harpadon nehereus* showed THQ risks, while no CR risks were measured in the studied fishes [29]. Pb concentration in the coastal areas of Bangladesh, such as Kuakata, Pathorghata, Pirojpur, and Cox’s Bazar showed higher human health risks in *Pampus argenteus* and *T*. *ilisha* than the riparian areas [32]. In addition, Pb showed health risks through the consumption of crustaceans [25]. The Malaysian fishes showed medium chronic risks; whereas, the seafood species showed higher chronic risks [7]. The beginning of THQ and CR risks through the consumption of fishes sourced via bioaccumulation of HMs from the external environment to the fish body. Our BAFs data suggests that HMs transferred from water to *T*. *ilisha* tissues especially via gills and deposited to the liver then spread evenly throughout the muscle tissues (see details between the size groups and HMs section below). Only Cr showed the size-related accumulation, while the rest of the HMs was not sized specific. However, the BAFs might be varied among species even within the different individuals of the same species [85]. For example, in the Meghna River estuary, average BAFs in the commercially important fishes including *T*. *ilisha* muscles were reported as Pb (1042.29) > Cr (1036.47) > Cd (832.77) > Cu (772) [26]. In *T*. *ilisha* muscles, the BAFs ranged between 484.84–1073.43 in assessed HMs Meghna estuary [26]. In Ganga River (India), the studied fishes were calculated as bio-accumulative and followed as liver > gills > muscles [42].

### 4.4. L-W Relationships of the Selected Classes (S1–S5) of T. ilisha

The biometric features (L-W relationships) were analyzed to correlate any links between L-W and HMs. The *R*^2^ value and HMs concentration of *T*. *ilisha* size did not follow any correlation. However, HMs accumulation, regression coefficient (b) and condition factor (CF) showed a similar positive correlation in the juvenile to the pre-adult stage (S1–S2) (Table S1) suggesting that the HMs accumulation, b and CF value were equally decreased with the increase of L-W. The *T*. *ilisha* specimens in the Tentulia river (Bangladesh) classified into 27.3, 65.87, 109.41, 227.95, 365.45, 491.24, 788.94, and 1089.35 g body weight, and the age was measured as, 0.29, 0.41, 0.55, 0.73, 0.94, 1.23, 1.70, and 2.65 years, respectively [83]. Besides, the *T*. *ilisha* specimens in different areas of Bangladesh including the Meghna river estuary ranged from 24.8 ± 5.52 to 45.9 ± 1.14 cm length and 178.3 ± 96.20 to 1378.1 ± 155.51 g body weight, which was determined between <1–6 years [71]. Moreover, the *T*. *ilisha* specimens were measured between 14–57 cm and 1–5 y in Kuwait and the *T*. *ilisha* ranged from 15– 50 cm and <1–5 y in Pakistan, respectively [86,87]. Considering the L-W parameters, our selected specimens correspond well between <1 to 5 y and might be classified as, S1 (~0.6), S2 (~1.0), S3 (~2.3), S4 (~ 4.0) and S5 (5.0) y, respectively. More than 90% *T*. *ilisha* corresponding to S4 group are popularly consumed in Bangladesh [24]. The calculated CF ranged from 1.9–2.5 among the studied *T*. *ilisha*. CF > 1.0 indicates that all the *T*. *ilisha* were collected from a healthier population. However, the L-W relationships of *T*. *ilisha* also depend on the availability of foods, seasons, health, habitats, and sexes [70,88,89,90]. For example, in Indian *T*. *ilisha*, an isometric growth was estimated during the monsoon, whereas negative allometric growth was measured during winter [89]. In addition, a positive allometric growth (b > 3.0) was calculated in *T*. *ilisha* from the Meghna estuary, Padma River, Tetulia River, and Bay of Bengal (Bangladesh). However, an isometric (b = 3.0) and negative allometric (b < 3) growth were determined in the Kali River and Gajlajur Haor (Bangladesh), respectively [88].

### 4.5. T. ilisha Classes (S1–S5) vs. HMs Influence

No consistent size-related trends were found among the assessed fish and HMs (Tables S3 and S4). The Pearson correlations and linear regressions showed randomly positive significant relations among the HMs and *T*. *ilisha* sizes (Tables S3 and S4). The degree of relationship between HMs and fish sizes depends on many factors. Habitats, feeding habits, swimming behavior, seasons, metabolic activity, properties of the water (physical and chemical) are responsible for the uptake and accumulation of HMs other than body size and weight of fish [9,47,48]. Habitats, such as the pelagic *T*. *ilisha* feeding with phytoplankton and zooplankton, uptake fewer HMs than the bottom feeder carnivorous/omnivorous *Otolithes ruber*, *H*. *nehereus* [29,47,48]. In addition, fishes like *T*. *ilisha* have a smaller trophic level and usually uptake a lower amount of HMs than the higher trophic *Trachurus trachurus* [8]. Moreover, the HMs concentration in *T*. *ilisha* was higher in Cox’s Bazar than Sundarbans and Bhola (Bangladesh), respectively [25]. Fluctuations of HMs are also influenced by the seasons [29]. Nevertheless, consistent and significantly positive tissue-type relations were found among the studied *T*. *ilisha* (Figure S3). HMs were highly accumulated in gills and liver; while HMs were not significantly accumulated in muscles because gills have direct contact with the external water. Moreover, the gill surface is negatively charged, supporting a possible direction for gill-HMs contact with positively charged HMs ions [10]. The HMs in the freshwater fish gills were determined higher than liver and muscles in the Tigris River, Baghdad, correspond to our findings [84]. HMs also deposit in the liver through the metal-binding proteins in animal tissues with oxygen carboxylate, amino groups, and the nitrogen-sulfur of the mercapto group in the metallothioneins [48,91]. This metallothionein group accelerates biosynthesis after exposure to sublethal levels of HMs [85]. Although few studies [45,46] reported higher HMs content in the *T*. *ilisha* liver than gills, they did not consider the sizes. As brood *T*. *ilisha* migrates from the ocean to fresh water for breeding, they expense their storage energy through metabolisms. As a result, there might be lower HMs content in the liver than gills. Continuous upwelling and mixing make the river and estuarine system more favorable for juvenile *T*. *ilisha* feeding more eutrophic [92]. Thus, the smaller *T*. *ilisha* intake higher HMs through feeding than the adult ones. On the other hand, muscles are not an active HMs binding site, and as a result, lower accumulation occurs with these tissues [93]. Muscles require lower concentrations of HMs during the enzymatic and oxidative reactions for the synthesis and usage of ATP, the production of intracellular proteases (calpains), and other endocrine activities [94]. In addition, fish skin has low permeability to HMs, and muscles constitute a more significant body proportion than other tissues, which allows the HMs to spread uniformly throughout the muscle tissues [85].

## 5. Conclusions

Only Cr (VI) exceeded all the recommended guidelines and followed the size-related trends. Cr (VI) might be considered as an industrial pollutant indicator metal in the Padma–Meghna River systems. In addition, HMs showed a significantly positive (*p* < 0.05) relationship in the gills and liver, but not in the muscles. Cr was calculated as bio-accumulative in the gills and liver, and presented acute non-carcinogenic health risks to consumers. However, no carcinogenic health risks were determined, except the largest size gills for children. Consumers in the studied regions could eat *T*. *ilisha* as the rest of the HMs showed no health risks. Our study suggests that HMs contents and accumulation are positively dependent on *T*. *ilisha* sizes, but not consistently. Nevertheless, consistently positive relations found among the tissue types. The variance among HMs and sizes might be species-specific that depends on habitats, food and feeding habits, metabolic activities, and properties of the water. However, examining bioaccumulation of HMs under laboratory conditions, sediments data, and wide scale repeated sampling in different seasons and precautionary actions are recommended as future perspectives.

## Figures and Tables

**Table 1:** Length-weight based size classes, habitat, and trophic level of *T. ilisha* for the purposes of this study.

Size Class	Length Range Min.—Max. (Average) ± SD cm	Weight Range Min.—Max. (Average) ± SD g	Habitats	Trophic Level
S1	19.1–20.5 (20.0) ± 0.5	100.1–105.3 (102.9) ± 1.5		
S2	25.1–26.6 (26.0) ± 0.4	175.2–180.3 (178) ± 1.6		Phytoplankton,
S3	29.0–30.8 (29.8) ± 0.6	245.1–251.3 (247.5) ± 1.8	Pelagic	zooplankton,
S4	38.5–40.5 (39.4) ± 0.6	700.1–710.1 (706.3) ± 2.8		plants, mollusks,
S5	45.0–46.5 (45.8) ± 0.5	1190.2–1200.4 (1194.2) ± 3.2		crustaceans

**Table 2:** Concentration (Mean ± SD) of heavy metals (µg g^−1^ dry weight) in muscle, liver, and gills of the five size classes (S1–S5) of *T. ilisha*. Comparatively, reported values are also available from the literature and guidelines. n/a = not available.

Organ	Size Class	Cu	Zn	Pb	Cd	Cr
	S1	9.12 ± 0.79	65.32 ± 5.2	0.013 ± 0.005	0.001 ± 0.000	7.60 ± 0.65
	S2	6.67 ± 0.540	49.56± 3.88	0.021± 0.004	0.003 ± 0.000	6.64 ± 0.56
Muscle	S3	15.52 ± 1.170	47.16 ± 3.75	0.015 ± 0.002	0.001 ± 0.003	12.08 ± 1.03
	S4	15.56 ± 1.41	61.32 ± 4.61	0.011 ± 0.001	0.008 ± 0.006	23.96 ± 2.43
	S5	4.76 ± 0.48	40.88 ± 3.39	0.014 ± 0.002	0.003 ± 0.000	5.44 ± 0.54
	S1	9.52 ± 0.92	63.4 ± 5.39	0.013 ± 0.002	0.004 ± 0.000	4.52 ± 0.48
	S2	23.48 ± 1.42	121.60 ± 10.7	0.017 ± 0.002	0.002 ± 0.000	172.32 ± 14.71
Liver	S3	11.12 ± 0.90	117.43 ± 8.75	0.013 ± 0.001	0.003 ± 0.000	7.61 ± 0.63
	S4	24.64 ± 1.62	108.76 ± 6.67	0.015 ± 0.002	0.004 ± 0.000	24.04 ± 2.02
	S5	23.68 ± 1.51	132.76 ± 11.08	0.014 ± 0.005	0.001 ± 0.000	15.88 ± 0.90
	S1	18.28 ± 1.61	189.04 ± 14.93	0.017 ± 0.001	0.019 ± 0.002	326.64 ± 19.58
	S2	17.84 ± 1.56	161.64 ± 11.47	0.086 ± 0.007	0.053 ± 0.004	48.76 ± 4.17
Gill	S3	11.24 ± 1.09	96.40 ± 6.51	0.011 ± 0.002	0.03 ± 0.003	76.44 ± 6.54
	S4	8.01 ± 0.99	148.36 ± 10.21	0.015 ± 0.001	0.129 ± 0.012	23.48 ± 1.98
	S5	25.04 ± 1.87	134.04 ± 9.99	0.016 ± 0.001	0.469 ± 0.02	49.00 ± 3.33
	Mean	14.97 ± 6.96	102.71 ± 46.07	0.0194 ± 0.019	0.049 ± 0.120	53.63 ± 7.09
Literature and guidelines						
Meghna estuary [25]		4.06	n/a	3.33	0.10	0.64
Meghna River, Narsingdi [27]		1.18	11.21	0.64	0.085	0.057
Karnaphuli River, Chittagong [29]		n/a	n/a	0.62	0.12	0.46
* FAO/WHO [73]		38.46–115.38 (10.0–30.0)	192.30–384.61 (50.0–100.0)	1.92 (0.5)	0.20 (0.05)	7.70 (2.00)
* New Zealand CEPA [74]		38.46– 384.61 (10.0 –100.0)	153.84–384.61 (40.0 –100.0)	7.70 (2.0)	3.84 (1.0)	3.84 (1.0)
* Bangladesh (MOFL) [75]		19.23 (5.0)	192.30 (50.0)	1.15 (0.30)	0.96 (0.25)	3.84 (1.0)

* The guidelines presented in wet wt. were converted into dry wt. with assuming an average 74% water present in tissues [50] and shown in parenthesis.

**Table 3:** Relative study of HMs concentration (µg mL^−1^) obtained from the surface water at the confluence of the Padma and Meghna rivers (present study), guidelines, and relevant literature. na = not analyzed, bdl = below detection limit.

Source	Cu (µg mL^−1^)	Zn (µg mL^−1^)	Pb (µg mL^−1^)	Cd (µg mL^−1^)	Cr (µg mL^−1^)	References
Padma–Meghna rivers confluence	0.058 ± 0.04	0.070 ± 0.005	0.002 ± 0.001	0.002 ± 0.001	0.035 ± 0.002	Present study
Meghna River, Narayanganj	na	0.036	bdl	0.00	0.035	[37]
Meghna River, Narshingdi	0.027	0.04	0.01	0.018	0.02	[38]
India	2.750	6.180	0.562	0.712	0.495	[42]
Bay of Bengal	0.119–0.192	na	0.01–0.694	0.002–0.01	na	[40]
Saudi Arabia	7.85 ± 1.52	3.58 ± 0.94	0.56 ± 0.13	0.17 ± 0.04	1.36 ± 0.37	[79]
USEPA WHO	0.05–2.0	3.0	0.01- 0.05	0.003–0.01	0.050–0.1	[80]; [76,77,78]

**Table 4:** Values of non-carcinogenic risks (THQ) and carcinogenic risks (CR) of different size classes of fish (*T. ilisha*) and their organs for adults (A) and children (C). Bold values indicate Hazard Index > 1, * = contained CR risks.

					THQ								CR		
Organs	Sizes	Cu (A)	Cu (C)	Zn (A)	Zn (C)	Pb (A)	Pb (C)	Cd (A)	Cd (C)	Cr (A)	Cr (C)	Pb (A)	Pb (C)	Cd (A)	Cd (C)
	S1	0.047	0.207	0.047	0.207	0.001	0.003	0.000	0.001	0.522	**2.305**	2.15 × 10^-8^	1.01 × 10^-7^	1.30 × 10^-^^6^	5.73 × 10^-6^
Muscles	S2	0.034	0.152	0.034	0.150	0.001	0.005	0.001	0.003	0.456	**2.014**	3.48 × 10^-8^	1.62 × 10^-7^	3.90 × 10^-6^	1.72 × 10^-5^
	S3	0.080	0.353	0.032	0.143	0.001	0.004	0.000	0.001	0.830	**3.664**	2.49 × 10^-8^	1.16 × 10^-7^	1.30 × 10^-6^	5.73 × 10^-6^
	S4	0.080	0.354	0.042	0.186	0.001	0.003	0.002	0.007	**1.646**	**7.268**	1.99 × 10^-8^	9.28× 10^-8^	1.04 × 10^-5^	4.59 × 10^-5^
	S5	0.025	0.108	0.028	0.124	0.001	0.004	0.001	0.003	0.374	**1.650**	2.32 × 10^-8^	1.08 × 10^-7^	3.90 × 10^-6^	1.72 × 10^-5^
	S1	0.049	0.217	0.044	0.192	0.001	0.003	0.001	0.004	0.311	**1.371**	2.15 × 10^-8^	1.01 × 10^-7^	5.19 × 10^-6^	2.29 × 10^-5^
	S2	0.121	0.534	0.084	0.369	0.001	0.004	0.000	0.002	**11.841**	**52.270**	2.82 × 10^-8^	1.31 × 10^-7^	2.60 × 10^-6^	1.15 × 10^-5^
Liver	S3	0.057	0.253	0.081	0.356	0.001	0.003	0.001	0.003	0.522	**2.305**	2.15 × 10^-8^	1.01 × 10^-7^	3.90 × 10^-6^	1.72 × 10^-5^
	S4	0.127	0.561	0.075	0.330	0.001	0.004	0.001	0.004	**1.652**	**7.292**	2.49 × 10^-8^	1.16 × 10^-7^	5.19 × 10^-6^	2.29 × 10^-5^
	S5	0.122	0.539	0.091	0.403	0.001	0.004	0.001	0.005	**1.091**	**4.817**	2.32 × 10^-8^	1.08 × 10^-7^	7.79 × 10^-6^	3.44 × 10^-5^
	S1	0.094	0.416	0.130	0.573	0.001	0.004	0.004	0.017	**22.445**	**99.081**	2.82 × 10^-8^	1.31 × 10^-7^	2.47 × 10^-5^	1.09 × 10^-4^
	S2	0.092	0.406	0.111	0.490	0.005	0.022	0.011	0.048	**3.351**	**14.791**	1.43 × 10^-7^	6.65 × 10^-7^	6.88 × 10^-5^	3.04 × 10^-4^
Gills	S3	0.058	0.256	0.066	0.292	0.001	0.003	0.006	0.027	**5.253**	**23.187**	1.82 × 10^-8^	8.51 × 10^-8^	3.90 × 10^-5^	1.72 × 10^-4^
	S4	0.041	0.182	0.102	0.450	0.001	0.004	0.027	0.117	**1.613**	**7.122**	2.49 × 10^-8^	1.16 × 10^-7^	1.68 × 10^-4^	7.40 × 10^-4^
	S5	0.129	0.570	0.092	0.407	0.001	0.004	0.097	0.427	**3.367**	**14.863**	2.49 × 10^-8^	1.16 × 10^-7^	6.09 × 10^-4^	* 2.69 × 10^-3^
	Mean	0.077	0.340	0.071	0.312	0.001	0.005	0.010	0.045	3.685	16.267	3.22 × 10^-8^	1.50 × 10^-7^	6.36 × 10^-5^	2.81 × 10^-4^

**Table 5:** Bioaccumulation factors of heavy metals in muscles, liver, and gills of the different size classes of *T*. *ilisha*. Bold is used to identify bio-accumulative condition.

Organ	Sizes	Cu	Zn	Pb	Cd	Cr
Mussel	S1	40.9	253.8	1.7	0.1	56.5
	S2	29.9	184.1	2.7	0.4	49.3
	S3	69.6	175.2	2.0	0.1	89.7
	S4	69.8	227.8	1.6	1.0	178.0
	S5	21.3	151.8	1.8	0.4	40.4
Liver	S1	42.7	235.5	1.7	0.5	33.6
	S2	105.3	451.7	2.2	0.3	**1280.1**
	S3	49.8	436.2	1.7	0.4	56.5
	S4	110.5	404.0	2.0	0.5	178.6
	S5	106.2	493.1	1.8	0.8	118.0
Gill	S1	81.9	702.1	2.2	2.5	**2426.5**
	S2	80.0	600.4	11.2	6.9	362.2
	S3	50.4	358.1	1.4	3.9	567.8
	S4	35.9	551.1	2.0	16.8	174.4
	S5	112.2	497.9	2.0	61.0	364.0
Total	Mean	67.1	381.5	2.5	6.4	398.4

## Supplementary Materials

The following are available online at https://www.mdpi.com/article/10.3390/toxics9120341/s1, Figure S1: PCA of HMs in Padma–Meghna River water (present study) and threshold levels reported in Bangladeshi water and other regions (Ind. water-Indian water; Saudi Ara.- Saudi Arabian water) and guidelines (WHO), Figure S2: Loading plot of rotated PCA (Principal Component Analysis) indicates that the gills (G) and liver (L) are highly affected by heavy metals than muscles (M). [size classes from S1–S5; e.g., S1-G = S1 sized gills of *T*. *ilisha*], Figure S3: Pearson correlation shows the gills and liver (μg g dry wt.^−1^) are positively significant, while, the muscles are non-significant, Table S1: Estimated statistical parameters and length-weight relationships of *T*. *ilisha* from the Padma–Meghna confluence, Table S2: Heavy metals hierarchy in different sizes of *T*. *ilisha* and their organs, Table S3: Pearson correlations of heavy metals (HMs) (μg g dry wt.^−1^) among the size classes and tissues of *T*. *ilisha*. HMs and sizes were randomly positively significant (*p* < 0.05). [S1-M = S1 size muscles; S1-L = S1 size liver; S1-G = S1 size gills], Table S4: Size-wise heavy metals concentration in different tissues of *T*. *ilisha* using linear regression. *Y* is the metal concentrations (µm g^−1^, dry weight) in different tissues and sizes of *T*. *ilisha*, and *x* is the total length (cm) of *T*. *ilisha*. [Among HMs vs. tissue type regression 21.75% was significant].
