# Supplementary material for: Heavy Metals in the Fish Tenualosa ilisha Hamilton, 1822 in the Padma–Meghna River Confluence: Potential Risks to Public Health
Source: Toxics. 2021 Dec 8;9(12):341. doi: 10.3390/toxics9120341 (PMC8708538; doi:10.3390/toxics9120341)
Supplement: Supplementary file 1 [file toxics-09-00341-s001.zip › toxics-1469058-supplementary.pdf]

toxics

MDPI

Supplementary Materials: Heavy Metals in the Fish *Tenualosa ilisha* Hamilton, 1822 in the Padma–Meghna River Confluence: Potential Risks to Public Health

Md. Jahangir Sarker, Md. Ariful Islam, Farhana Rahman and Md Anisuzzaman

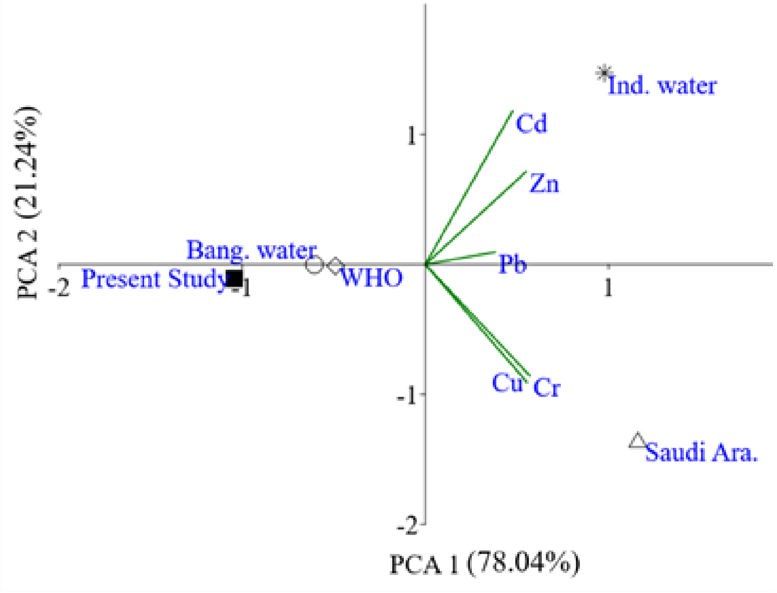

Figure S1. PCA of HMs in Padma-Meghna River water (present study) and threshold levels reported in Bangladeshi water and other regions (Ind. water-Indian water; Saudi Ara.- Saudi Arabian water) and guidelines (WHO).

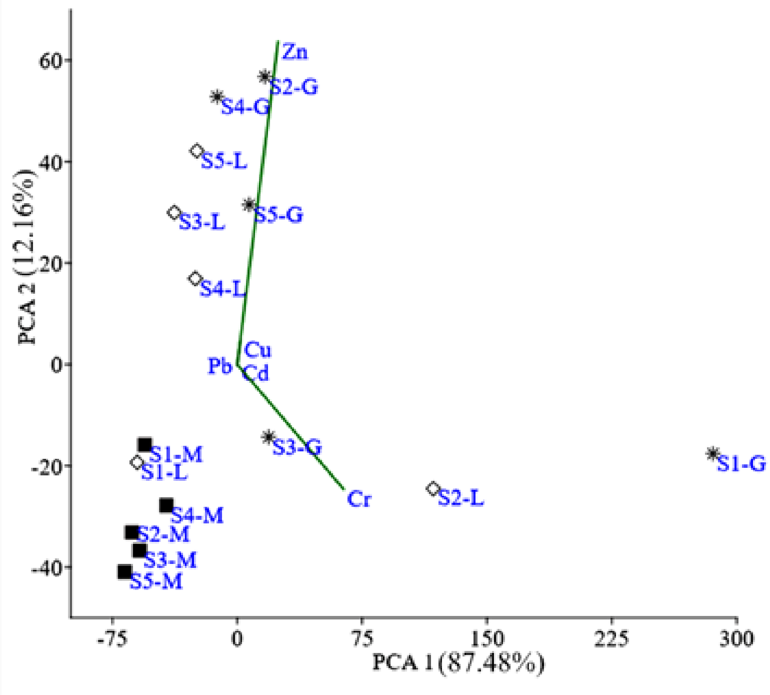

Figure S2. Loading plot of rotated PCA (Principal Component Analysis) indicates that the gills (G) and liver (L) are highly affected by heavy metals than muscles (M). [size classes from S1-S5; e.g. S1-G = S1 sized gills of hilsa].

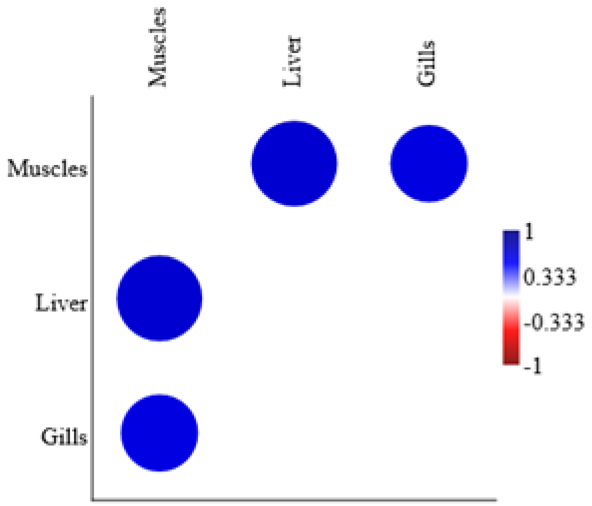

Figure S3. Pearson correlation shows the gills and liver (μg g dry wt.$^{-1}$) are positively significant, while, the muscles are non-significant.

Table S1. Estimated statistical parameters and length-weight relationships of *T. ilisha* from the Padma-Meghna confluence.

Study area	*T. ilisha* size	equation	*n*	Intercept (a)	Slope (b)	*p*-value	95% CL of a	95% CL of b	St. Err.	CF	R^2	References
Padma-Meghna River confluence	Overall	$W = aL^{3.02}$	125	0.02	3.02	< .05	0.00–0.01	2.94–3.09	0.06	2.2	0.99	Present study
	S1	$W = aL^{0.58}$	25	18.25	0.52	< .05	13.3–25.01	0.47–0.68	0.00	2.3	0.85	
	S2	$W = aL^{0.32}$	25	63.5	0.32	< .05	30.6–132	0.09–0.54	0.00	2.0	0.27	
	S3	$W = aL^{0.30}$	25	88.5	0.30	< .05	65.2–120.2	0.21–0.4	0.00	1.9	0.68	
	S4	$W = aL^{0.14}$	25	427.5	0.14	< .05	314.54–581	0.05–0.06	0.00	2.4	0.34	
	S5	$W = aL^{0.22}$	25	521.93	0.22	< .05	436.1–624.6	0.17–0.26	0.00	2.5	0.80	
Meghna estuary	35cm–500g	$W = aL^{3.2}$	-*not found	-	3.2	< .01	-	-	-	-	0.95	[84]
Padma River	35cm–476g	$W = aL^{3.42}$	-	-	3.42	< .01	-	-	-	-	0.97	
Bay of Bengal	37cm–582g	$W = aL^{3.2}$	-	-	3.2	< .01	-	-	-	-	0.93	
Tetulia River	37.5cm–751 g	$W = aL^{3.5}$	-	-	3.5	< .01	-	-	-	-	0.97	
Kali River	26cm–211.5	$W = aL^{3.0}$	-	-	3.0	< .05	-	-	-	-	0.98	
Gaglajur Haor	20cm–93g	$W = aL^{2.96}$	-	-	2.96	< .05	-	-	-	-	0.93	
Gujarat, India	35.4cm–626g	$W = aL^{3.07}$	270	6×10^{-7}	3.07	< .05	0.00	3.03–3.11	-	-	0.99	[85]
Gujarat, India	37cm–537.4	$W = aL^{2.76}$	42	0.02	2.76	< .05	0.00–0.08	2.42–3.11	-	-	0.93	[85]

Table S2. Heavy metals hierarchy in different sizes of *T. ilisha* and their organs.

Metal/organ/factors	Concentration order
Cr	S1 > S2 > S3 > S4 > S5
Cu	S5 > S4 > S2 > S3 > S1
Zn	S2 > S1 > S4 > S5 > S3
Pb	S2 > S1 = S4 = S5 > S3
Cd	S5 > S4 > S2 > S3 > S1
Muscles	S4 > S1 > S3 > S2 > S5
Liver	S2 > S4 > S3 > S1 > S5
Gills	S1 > S2 > S5 > S3 > S4
Muscles	Zn > Cr > Cu > Pb > Cd
Liver	Zn > Cr > Cu > Pb > Cd
Gills	Zn > Cr > Cu > Cd > Pb
R^2	S1 > S5 > S3 > S4 > S2
CF	S5 > S4 > S1 > S2 > S3
b	S1 > S2 > S3 > S5 > S4

HMs average total	S1 > S2 > S4 > S5 > S3

Table S3. Pearson correlations of heavy metals (HMs) (µg g dry wt.$^{-1}$) among the size classes and tissues of T. ilisha. HMs and sizes were randomly positively significant ($p < .05$). [S1-M = S1 size muscles; S1-L = S1 size liver; S1-G = S1 size gills].

	**S1-M**	**S2-M**	**S3-M**	**S4-M**	**S5-M**	**S1-L**	**S2-L**	**S3-L**	**S4-L**	**S5-L**	**S1-G**	**S2-G**	**S3-G**	**S4-G**	**S5-G**
**S1-M**		0.00	0.00	0.01	0.00	0.00	0.41	0.00	0.00	0.00	0.53	0.00	0.12	0.00	0.01
**S2-M**	1.00		0.00	0.01	0.00	0.00	0.39	0.00	0.00	0.00	0.50	0.00	0.11	0.00	0.01
**S3-M**	0.97	0.98		0.00	0.01	0.01	0.32	0.01	0.00	0.00	0.44	0.01	0.09	0.01	0.00
**S4-M**	0.96	0.96	0.98		0.01	0.01	0.19	0.02	0.00	0.01	0.28	0.00	0.03	0.01	0.00
**S5-M**	1.00	1.00	0.97	0.96		0.00	0.38	0.00	0.00	0.00	0.50	0.00	0.11	0.00	0.01
**S1-L**	1.00	1.00	0.97	0.95	1.00		0.45	0.00	0.00	0.00	0.58	0.01	0.15	0.00	0.01
**S2-L**	0.48	0.50	0.56	0.70	0.51	0.44		0.45	0.32	0.41	0.00	0.23	0.02	0.35	0.20
**S3-L**	1.00	1.00	0.96	0.94	1.00	1.00	0.45		0.00	0.00	0.57	0.01	0.14	0.00	0.01
**S4-L**	0.99	0.99	0.99	0.98	0.99	0.99	0.56	0.98		0.00	0.44	0.00	0.09	0.00	0.00
**S5-L**	1.00	1.00	0.98	0.96	1.00	1.00	0.48	1.00	0.99		0.53	0.00	0.13	0.00	0.01
**S1-G**	0.38	0.40	0.46	0.60	0.41	0.34	0.99	0.35	0.46	0.38		0.32	0.05	0.45	0.28
**S2-G**	0.98	0.98	0.97	0.99	0.98	0.97	0.65	0.97	0.99	0.98	0.56		0.04	0.00	0.00
**S3-G**	0.77	0.79	0.81	0.91	0.79	0.74	0.93	0.75	0.82	0.77	0.88	0.89		0.09	0.03
**S4-G**	0.99	1.00	0.96	0.96	1.00	0.99	0.54	0.99	0.98	0.99	0.45	0.99	0.82		0.00
**S5-G**	0.97	0.97	0.98	1.00	0.97	0.95	0.69	0.95	0.99	0.97	0.60	1.00	0.91	0.97	

Table S4. Size-wise heavy metals concentration in different tissues of *T. ilisha* using linear regression. *Y* is the metal concentrations (µm g^{-1}, dry weight) in different tissues and sizes of *T. ilisha,* and *x* is the total length (cm) of hilsa. [Among HMs vs tissue type regression 21.75% was significant].

**tissues**	**size**	**data**	**Cu**	**Zn**	**Pb**	**Cd**	**Cr**
Muscles	S1	DF	24	24	24	24	24
		equation	$Y = 0.139x+6.341$	$Y = 0.330x+61.7$	$Y = -7.0\times10^{-4}x+0.027$	$Y = 8.0\times10^{-4}x-0.014$	$Y = -0.058x+8.757$
		R^2	0.02	0.08	0.00	0.05	0.00
		p value	0.2	0.16	0.70	0.33	0.75
	S2	DF	24	24	24	24	24
		equation	$Y = -0.014x+7.048$	$Y = -0.228x+55.466$	$Y = 0.003x-0.048$	$Y = 9.0\times10^{-4}x-0.019$	$Y = -0.023x+7.256$
		R^2	0.00	0.00	0.41	0.16	0.00
		p value	0.97	0.72	0.01	0.09	0.93
	S3	DF	24	24	24	24	24
		equation	$Y = 0.787x-7.922$	$Y = 1.285x+8.906$	$Y = 0.001x-0.022$	$Y = 3.0\times10^{-4}x-0.007$	$Y = 1.216x-24.109$
		R^2	0.63	0.58	0.04	0.04	0.61
		p value	0.00	0.00	0.52	0.38	0.00
	S4	DF	24	24	24	24	24
		equation	$Y = 0.271x+4.870$	$Y = 1.068x+19.189$	$Y = 0.002x-0.080$	$Y = 1.3\times10^{-3}x-0.042$	$Y = 1.244x-25.12$
		R^2	0.01	0.05	0.26	0.36	0.39
		p value	0.79	0.59	0.02	0.00	0.04
	S5	DF	24	24	24	24	24
		equation	$Y = -0.114x+9.992$	$Y = 0.685x+9.470$	$Y= 0.003x-0.152$	$Y = 0.003x-0.139$	$Y = 0.9x-35.781$
		R^2	0.02	0.05	0.53	0.37	0.36
		p value	0.20	0.73	0.00	0.00	0.00
Liver	S1	DF	24	24	24	24	24
		equation	$Y = -0.192x+13.399$	$Y = -0.048x+64.423$	$Y = 0.001x-0.012$	$Y = 0.0005x-0.006$	$Y = 0.031x+3.894$
		R^2	0.02	0.00	0.03	0.02	0.00
		p value	0.44	0.85	0.69	0.677	0.43
	S2	DF	24	24	24	24	24
		equation	$Y = 0.010x+23.346$	$Y = 0.566x+106.89$	$Y = 0.005x-0.114$	$Y = 3.0\times10^{-5}x+0.002$	$Y = -0.057x+173.1$
		R^2	0.00	0.017	0.68	0.32	0.12
		p value	0.98	0.52	0.00	0.08	0.70
	S3	DF	24	24	24	24	24
		equation	$Y = 0.744x-11.034$	$Y = 2.027x+56.977$	$Y = -8.0\times10^{-6}x+0.014$	$Y =-1.0\times10^{-4}x+0.007$	$Y = 1.386x-33.65$

		R^2	0.37	0.51	0.00	0.002	0.68
		p value	0.07	0.00	0.96	0.62	0.00
	S4	DF	24	24	24	24	24
		equation	$Y = -0.764x+54.785$	$Y = 1.128x+64.274$	$Y = 0.001x-0.037$	$Y = 0.002x-0.094$	$Y = 1.123x-20.276$
		R^2	0.09	0.16	0.35	0.40	0.30
		p value	0.12	0.04	0.02	0.00	0.165
	S5	DF	24	24	24	24	24
		equation	$Y = -0.188x+32.342$	$Y = 3.854x-43.908$	$Y = 0.004x-0.160$	$Y = 0.003x-0.117$	$Y = 0.791x-20.389$
		R^2	0.02	0.42	0.46	0.48	0.17
		p value	0.45	0.00	0.00	0.00	0.23
	S1	DF	24	24	24	24	24
		equation	$Y = -0.021x+18.707$	$Y = 0.680x+175.43$	$Y = 8.0\times10^{-4}x+9.0\times10^{-4}$	$Y = 0.004x-0.057$	$Y = 1.906x+288.52$
		R^2	0.00	0.07	0.00	0.13	0.00
		p value	0.94	0.19	0.71	0.18	0.88
	S2	DF	24	24	24	24	24
		equation	$Y = -0.009x+17.966$	$Y = -0.439x+173.09$	$Y = 0.009x-0.153$	$Y = -1.0\times10^{-4}x + 0.055$	$Y = 0.053x+49.041$
		R^2	0.00	0.01	0.71	0.03	0.17
		p value	0.92	0.52	0.00	0.34	0.32
	S3	DF	24	24	24	24	24
		equation	$Y = 1.048x-19.934$	$Y = 1.745x+44.501$	$Y = 0.002x -0.050$	$Y = 0.005x-0.136$	$Y = 2.205x+10.805$
		R^2	0.46	0.63	0.18	0.30	0.76
		p value	0.00	0.00	0.08	0.01	0.00
	S4	DF	24	24	24	24	24
Gills		equation	$Y = 0.1x-4.885$	$Y = 2.398x+53.788$	$Y = 0.002x-0.075$	$Y = 0.008x-0.1297$	$Y = -0.048x+24.11$
		R^2	0.00	0.41	0.43	0.40	0.52
		p value	0.68	0.02	0.00	0.06	0.00
	S5	DF	24	24	24	24	24
		equation	$Y = 0.364x+8.340$	$Y = 1.875x+48.104$	$Y = 0.003x-0.144$	$Y = 0.055x-2.048$	$Y = 2.805x-78.546$
		R^2	0.04	0.31	0.54	0.21	0.42
		p value	0.61	0.08	0.00	0.05	0.02
